# Exogenous Oxytocin Administration Restores Memory in Female APP/PS1 Mice

**DOI:** 10.3233/JAD-230657

**Published:** 2023-11-21

**Authors:** Philippos Koulousakis, Emily Willems, Melissa Schepers, Ben Rombaut, Jos Prickaerts, Tim Vanmierlo, Daniel van den Hove

**Affiliations:** a Department of Psychiatry and Neuropsychology, School for Mental Health and Neuroscience, Maastricht University, Maastricht, The Netherlands; b Department of Neuroscience, Biomedical Research Institute, Faculty of Medicine and Life Sciences, Hasselt University, Hasselt, Belgium

**Keywords:** Alzheimer’s disease, APPswePS1dE9, object location task, oxytocin

## Abstract

**Background:**

Current treatment options for Alzheimer’s disease (AD) are limited, inefficient, and often have serious side effects. Oxytocin is a neuropeptide implicated in a variety of central processes, such as social and reproductive behaviors. Among others, it has garnered attention in various domains of psychiatric research, while its role in the development and course of neurodegenerative disorders like AD is rather unknown.

**Objective:**

This study aimed to investigate the role of exogenous oxytocin administration on memory, specifically in view of AD, as a potential novel treatment option.

**Methods:**

We describe a novel treatment approach by using a relatively low dose of long-term intranasal oxytocin treatment, to restore memory deficits in female APPswePS1dE9 mice.

**Results:**

Female APPswePS1dE9 mice treated with oxytocin showed increased spatial memory performance in the object location task and improved working memory in the Y-Maze, while indicating decreased sociability.

**Conclusions:**

These results indicate that oxytocin is able to reverse acquired cognitive deficits in female APPswePS1dE9 mice.

## INTRODUCTION

Alzheimer’s disease (AD) is a fatal progressive neurodegenerative disease, characterized by accumulation of extracellular amyloid plaques, intracellular neurofibrillary tangles, and neuronal loss, leading to cognitive impairment [1]. AD is the leading cause of dementia, with a current prevalence of over 50 million people, which is expected to increase to over 152 million by 2050 [2]. AD is a multi-factorial disease comprised of genetic, epigenetic, and environmental risk factors [3]. It is classified into sporadic and familial AD, with the latter pertaining to genetic risk factors of dominantly inherited mutations within risk genes, such as in the *PSEN1*, *PSEN2*, *APOE*, and *APP* genes [4, 5]. While familial cases of AD are rare, sporadic AD makes up the largest amount of cases and does not have a clear genetic variation underlying disease onset [6]. One of the main challenges with AD remains that symptoms appear years after disease onset, leading to late and inefficient treatment [7].

Main treatment options available include acetylcholinesterase inhibitors, such as donepezil, galantamine, and rivastigmine, and N-methyl D-asparate receptor antagonists, such as memantine [8]. More recently, aducanumab, which has been suggested to decrease cognitive decline by removing accumulated amyloid-β42 (Aβ_42_), has been indicated as a potential treatment for AD [9]. However, aducanumab has been heavily criticized, as literature suggests only a weak correlation between Aβ_42_ accumulation and cognitive decline, and other anti-Aβ_42_ treatments have broadly failed to meet clinical endpoints in phase III trials [10– 13]. While the various treatment options aid patients for a limited amount of time, they are often accompanied by severe side effects such as nausea, headaches, and insomnia [14, 15]. On top of aforementioned side effects, these treatment options only temporarily slow down disease progression, while not improving the condition of the patient outside of mild symptom alleviation [13]. Ultimately, quality of life decreases drastically, indicating the need for new treatment options.

Oxytocin is a neuropeptide involved in a wide variety of mechanisms, such as lactation, social behavior, stress regulation, appetite, and associative learning [16, 17]. It is primarily produced in the hypothalamic paraventricular (PVN) and supraoptic (SON) nuclei [17]. Oxytocin has been shown to stimulate hippocampal neurogenesis via oxytocin receptors (OXTR) expressed in CA2 and CA3 pyramidal neurons [18]. The OXTRi is mainly viewed as a Gq/11 coupler, stimulating phospholipase Cβ (PLCβ) activity causing phosphoinositide accumulation and intracellular Ca^2 +^ elevation [19]. When oxytocin binds to the OXTR, the PLCβ pathway is activated, leading to transactivation of the epidermal growth factor receptor (EGFR) and activation of phosphatidylinositol 3-kinases (PI3K) as well as extracellular signal-regulated kinase 1/2 (ERK1/2) [20]. This activation increases dendritic protein kinase m zeta (PKM*ξ*) protein synthesis via mammalian target of rapamycin (mTOR) signaling [21]. PKM*ξ* is associated with maintenance of late-phase long term potentiation (LTP), a key process in hippocampal memory formation [22].

Recently, *oxt*, encoding for oxytocin, was shown to be differentially methylated in AD both within several brain regions as well as in the blood in several independent cohorts [23, 24]. DNA methylation is an epigenetic mechanism, regulating gene expression dependent on the exact location and degree of methylation of a gene [25]. These findings on differential methylation in AD mark *oxytocin* as a potential early biomarker and treatment angle for AD, the latter of which is even more interesting, with oxytocin being a Food and Drug Agency (FDA)-approved drug for aiding facilitation of labor in antepartum to strengthen uterine contractions and postpartum to deliver the placenta and control postpartum hemorrhage [26].

It is interesting to note that human studies investigating the effects of oxytocin, for instance on schizophrenia and autism, are plenty [27]. However, there is currently no human study investigating the role of oxytocin to treat memory loss. In fact, studies have shown that a single dose intranasal oxytocin administration (24 IU) leads to selective amnesia in healthy humans [28]. Notably, low-dose administration of oxytocin recently has been tested as a potential treatment avenue for AD in mice and rats [29, 30].

The present study investigates the potential role of low-dose oxytocin administration as a treatment for AD, by making use of the APPswePS1dE9 mouse model as well as *in vitro* models. The APPswePS1dE9 model shows Aβ_42_ accumulation from 4– 6 months and cognitive deficits from 7 months of age [31]. We therefore investigated the effects of long-term oxytocin treatment on Aβ_42_ plaque load as well as memory loss using female APPswePS1dE9mice.

More specifically, 8-month-old APPswePS1dE9mice displaying memory loss were treated intranasally with 0.5*μ*g/kg oxytocin for 42 days. The effects of oxytocin on memory were assessed using the Object Location Task (OLT) and Y-Maze test. We further investigated sociability. We also examined the potential mechanistic action of the treatment by investigating Aβ_42_ accumulation using immunohistochemistry, as well as by assessing mTOR signaling through qPCR and assessing neurite growth *in vitro*.

## MATERIALS AND METHODS

### Animals

Male heterozygous APPswePS1dE9 mice were backcrossed with wildtype (WT) female C57Bl6/J mice to obtain heterozygous APPswePS1dE9 mice and WT littermates. Animals were housed in the conventional animal facility at BIOMED, Hasselt University (Hasselt, Belgium). Female offspring were housed individually three weeks prior to behavioral training in standard cages on sawdust bedding in an air-conditioned room (22°C) and were exposed to an inverse 12 h light/12 h dark cycle. Animals had *ad libitum* access to food and water. All experiments were performed in accordance with the ethical committee for animal experiments of Hasselt University (National Identification Code: 202039).

### Behavioral setup

At the onset of the *in vivo* experiment, mice were eight months old and split into four treatment groups. Allocation of animals into oxytocin or vehicle groups was randomized, based on cognitive performance to ensure all groups had comparable d2 scores pre-treatment. Two groups (APPswePS1dE9 *n* = 8 (AD OXT); WT *n* = 10 (WT OXT)) received a dose of 0.5*μ*g/kg oxytocin (European Pharmacopoeia Reference Standard, O0700000) intranasally, twice a day, for 42 days in total. The other groups (APPswePS1dE9 *n* = 8 (AD CTL); WT *n* = 10 (WT CTL) received vehicle (PBS, Lonza, BE17-516F) during that same period. One dose was administered in the morning, the second one in the evening. Briefly, animals were manually fixated and received oxytocin by administering 10*μ*l of oxytocin to both nostrils. In order to exclude acute effects of the administration, on days where behavior was being assessed, animals were treated only once, after performing the behavioral experiment.

### Genotyping

DNA was extracted from tail tissue by incubation in an extraction mix containing 10% 10x KAPA express extract buffer (KAPA biosystems, KK7101), 2% 1U/*μ*l.KAPA express extract enzyme (KAPA biosystems, KK7101), and 88% milliQ, for 10 min at 75°C, and subsequently 5 min at 95°C. For the PCR, a master mix containing 50% 2x KAPA2 G fast genotyping mix with green dye (KAPA biosystems, KK5621), 5% 10*μ*M forward primer (5’- CCG AGA TCT CTG AAG TGA AGA TGG ATG -3’), 5% 10*μ*M reverse primer (5’- GTG GAT ACC CCC TCC CCC AGC CTA GAC C -3’), and 40% MilliQ, was used. The PCR amplification program was implemented on the BIO-RAD T100 thermal cycler and included 35 cycles (95°C 15 s; 60°C 15 s; 72°C 15 s) after an initial denaturation step of 3 min at 95°C. PCR products were analyzed on a 1% agarose gel along with a 100 bp DNA ladder (Invitrogen) and visualized using the LI-COR biosciences D-Digit gel scanner.

### Behavioral assessment

Animals were initially handled, weighed, and habituated in the arena for a week prior to testing. OLT was performed as previously described [32– 34]. Animals were assessed prior to treatment and after 28 days of treatment. Briefly, two identical objects were placed symmetrically in the middle of the arena. Five min before trial 1, animals were put in an empty cage to stimulate interest. During trial 1, a mouse was put into the arena by facing it towards the wall and letting it explore for 4 min, then returning it to its home cage. One hour after trial 1, the animal was put back into the arena for 4 min and allowed to explore the identical objects again. This time one of the objects had moved in position in the arena. The change of location was randomized across treatment conditions to reduce potential bias. Furthermore, four different sets of objects were used that were randomized across groups to reduce preference bias towards a certain object (Supplementary Figure 1). Animals were scored on their ability to recognize the object that had moved in trial 2 to assess spatial memory. The time spent exploring an object was measured in both trials and a discrimination index was calculated based of the total amount of exploration of the objects and the exploration of the newly moved object.

Animals were assessed in the Y-maze after 29 days of treatment (Supplementary Figure 5). In the Y-maze, animal’s alterations were scored for 6 min to assess working memory. Mice were placed centrally into the Y-maze and were free to explore the arena for 6 min. Each entry into an arm was recorded. To assess working memory of the mouse, the percentage of correct alternations made were calculated. This was achieved by dividing the number of correct triads (a series of three choices, composed of three subsequent different arm entries) by the maximum possible alternations (total number of entries minus 2) multiplied by 100. If the score was significantly above 50%, it indicated that the animal had functional working memory, as this was higher than the chance level for choosing the unfamiliar arm.

After 30 days of treatment, sociability was assessed by using a rectangular maze with 3 distinct areas (20×16×25 cm). The side areas had a small circular bar cage in them which was either empty or contained a female WT animal that was not part of the study cohort. Animals were placed in the center area and allowed to explore for 10 min. Time spent in the same area as the WT over the empty area was measured (Supplementary Figure 6).

### General tissue processing

12 days after finalizing behavior tests, mice were sacrificed by transcardial perfusion with PBS/1% heparin (LEO, 5000 IU/ml) after a lethal intraperitoneal injection with Dolethal (200 mg/kg). Brains were hand-dissected and divided into the left and right hemisphere. The left hemisphere was used to manually dissect the hippocampus and frontal cortex, which were snap-frozen in liquid nitrogen. AD hippocampi were used to investigate plaque load using an Aβ_42_ ELISA. WT hippocampi were used for the oxytocin ELISA. The right hemisphere was fixated in 4% paraformaldehyde overnight and sucrose-infiltrated (10%, 20%, and 30%) for cryopreservation. The fixated hemispheres were mounted in frozen section comp 22 (FSC22) and frozen in ice-cold isopentane. Afterwards, the mounted hemispheres were cut into 10*μ*m-thick sagittal slices on a cryostat and mounted on glass slides. Cryocoupes were stored at room temperature (RT) until further use.

### Primary rat neuronal cell culture

Cortices from postnatal P0-P1 rat pups were dissected in 9 ml 37°C pre-warmed HBSS (Gibco, 14175-095) with 7 mM HEPES buffer solution (Gibco, 15630-049) and 100 U/ml Pen/Strep (Sigma, P4333). Tissue was dissociated by 15 min incubation with 1 ml trypsin 1x in HBSS/HEPES 10x, followed by washing three times in MEM (Gibco, 31095- 029) with 10% heat-inactivated horse serum (Gibco, 26050-088), 0.6% glucose, and 100 U/ml Pen/Strep. Finally, tissue was completely homogenized by mechanical dissociation. Cell suspension was filtered over a 70*μ*m cell strainer (Greiner, 5420070), and the pellet was resuspended. Cells were plated at a density of 5000 cells per well in a 96 well plate. 24 h later, the MEM/horse serum medium was replaced by neurobasal medium (Gibco, 21103-049) with 1x B27 (Gibco, 17504-044), 2 mM L-glut (Sigma, G7513), and 100 U/ml Pen/Strep to ensure neuronal survival over glial cell survival. Next, oxytocin was added in concentrations of 0.5*μ*M (*n* = 9), 1*μ*M (*n* = 9), and 2*μ*M (*n* = 9). PBS was used as a negative control. The 96-well plate was incubated in the IncuCyte (Essen Bioscience) at 5% CO2 for five days, where a camera took pictures in a time-lapse manner, of the same area of each well through a 10x objective every 3 h. Neurons received a boosting dose of oxytocin after 48 h. Data analysis was performed with the IncuCyte NeuroTrack software module.

### RNA isolation and real-time qPCR

Hippocampal RNA was isolated using qiazol/chloroform and the RNeasy Mini kit (Qiagen, 74104). cDNA was synthesized using qScript cDNA SuperMix 6 (Quanta bio). Real-time qPCR was completed on the QuantStudio 3 (Applied Biosystems) using a master mix containing 67% SYBR green (Applied Biosystems), 25% MilliQ, 4% 10*μ*M forward primer, and 4% 10*μ*M reverse primer. Cycling parameters were 95°C for 20 s followed by 40 cycles of 95°C (3 s), and 60°C (30 s), ending with a melt curve stage of 95°C (15 s), 60°C (30 s), and 95°C (15 s). Primer sequences and used housekeeping genes are listed in the supplementary tables. The relative quantification of gene expression was accomplished using the *ΔΔ*Ct method.

### Aβ immunohistochemistry

Sagittal sections of the left hemisphere were stained for Aβ_42_ to assess plaque load in cortex as well as hippocampus. Endogenous peroxidases were blocked by incubation with 3% H_2_O_2_ in methanol at RT. Slides were washed three times in 1x TBS, 0.3% Triton X-100 and blocked with 5% bovine serum albumin (Sigma, A7906) and 1% goat serum in 1x TBS for 1 h at RT. Primary mouse anti-amyloid beta antibody (Millipore, clone WO-2) was applied in a 1:8000 dilution and incubated overnight at 4°C. After washing five times, slides were incubated with the EnVision+ Dual Link System-HRP kit (Dako, K4061) for 30 min at RT. A three-part washing step was performed and diaminobenzidine (DAB, Dako, K3468) was applied for 1 min to develop the slides. Slides were washed three times and counterstained with hematoxylin (Leica, 3801582E) for 1 min. Cryosections were rinsed under running tap water and dehydrated in 70% (2 min) and 100% ethanol (2 min). Slides were cleared in xylene for 1 min and mounted using DPX (Merck) in a 1/10 dilution in xylene. The amount of Aβ_42_ were quantified in the cornu amonis 1 (CA1) and 3 (CA3) in 5 sections. Digital images were obtained using a Leica DM 2000 LED microscope (20x objective, Leica Microsystems). The amyloid plaque load was quantified by using the analyze particles function in Fiji ImageJ.

### Aβ phase separation

Serial extraction of Aβ in hippocampal tissue was performed by generating four Aβ phases. Tris-buffered saline (TBS) (pH = 7.2), 0.1% Triton X-100 buffer, 2% sodium dodecyl sulfate (SDS) buffer, and 70% formic acid buffer were used. The buffers created the extracellular soluble fraction, intracellular soluble fraction, membrane associated fraction, and insoluble fraction, respectively [35]. Brain tissue or homogenates were sonicated twice for 30 s in 1 ml buffer per 150 mg wet weight of brain tissue using the S40 Elmasonic (Elma). Each fraction was obtained upon supernatant collection after centrifugation at 4°C and 17000 g. For the first, second and third fraction, the samples were centrifuged for 20 min. For the fourth fraction, the samples were centrifuged for 60 min. Fractions were stored at – 80°C.

### Bicinchoninic acid (BCA) assay

The total protein concentration (mg/ml) was determined via the BCA protein assay using the extracellular soluble fractions and the Pierce BCA Protein Assay Kit (Thermo Scientific). Sample fractions were diluted in 1x TBS buffer (1:40), as well as bovine serum albumin (BSA) standards. Upon addition of 200*μ*l working solution in each well, the reaction was initiated. After 30 min of incubation, the absorbance of each well could be measured at 570 nm using the Clariostar Plus plate reader (Isogen Life Science).

### ELISA

A human Aβ_42_ sandwich ELISA (Invitrogen product number: KHB3441) was used to determine the membrane-associated Aβ_42_ and insoluble Aβ_42_ concentration (pg/ml). Sample fractions were diluted in Standard diluent buffer (1:40). The Aβ_42_ standard (2000 pg/ml) was reconstituted with deionized water and diluted in standard diluent buffer to become several standards. After adding standards, controls and samples into the wells, a human Aβ_42_ detection antibody was added, and the ELISA plate was incubated while shaking for 3 h. After sequentially adding anti-rabbit IgG HRP, stabilized chromogen, and stop solution to each well, with incubation steps in between, the absorbance (at 450 nm) was read using the Clariostar Plus plate reader (Isogen Life Science).

An oxytocin colorimetric competitive enzyme immunoassay ELISA (Enzo Life Sciences product number: ADI-900-153A-0001) was applied to determine the amount of oxytocin in the hippocampus (pg/ml). Volume of homogenate was adjusted to the wet weight of samples. WT hippocampi were diluted in Standard diluent buffer (1:20). The oxytocin standard was reconstituted with deionized water and diluted according to the manufacturer’s protocol. After adding standards, controls, and samples into the wells. Conjugate was added to each well except the total activity control well, followed by adding the oxytocin antibody. Plate was sealed and incubated at 4°C for 24 h. After washing according to manufacturing protocol, oxytocin conjugate was added and pNpp substrate was added to each well incubating at room temperature for 1 h. Finally, stop solution was added and the plates’ optical density was read immediately using the Clariostar Plus plate reader (Isogen Life Science) at 405 nm.

### Statistical analysis

Statistical analysis was performed using Graphpad PRISM version 9.2.0. OLT and sociability were assessed using Two-way ANOVA to investigate treatment effect of oxytocin for both WT and AD animals Furthermore, one-sample *t*-tests against 0 were performed to test spatial memory in the OLT. In the Y-maze, we assessed the animals’ performance against chance level (50%) using a one-sample *t*-test. We based our power calculations on a previous study conducted in our lab [31]. G-power V.3.1.9.4 was used to determine sample size (*n* = 36, *p* = 0.05, power 0.80).

## RESULTS

### Intranasal oxytocin administration reverses memory loss in female APPswePS1dE9 mice

**Fig. 1:**
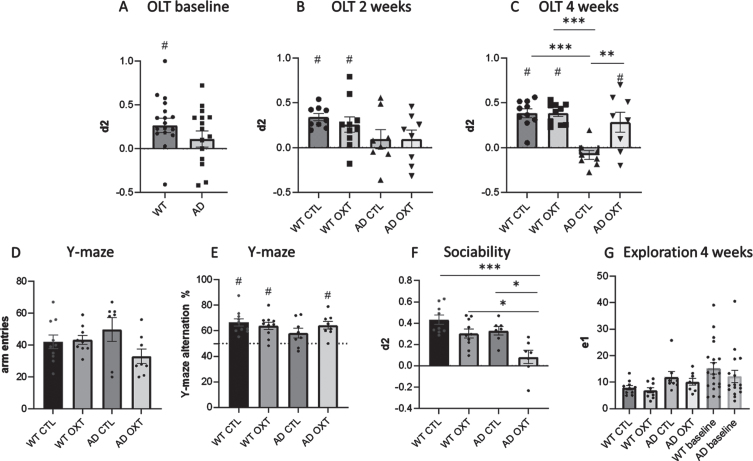
Intranasal administration of oxytocin reduces cognitive decline in an AD mouse model. Cognitive ability was evaluated using the OLT (A-C) and the Y maze spontaneous alternations task (D). For the OLT, the interval between the first (T1) and second (T2) trial was set at 1 h. The d2 value is calculated as the ratio between exploration time spent at moved object and the stationary object in T2. A d2 value of > 0 indicated intact object memory. At baseline, WT animals (*n* = 10) were able to distinguish between new and old location (A). After 2 weeks of treatment, WT animals in both conditions are able to distinguish between new and old location of objects, whereas AD animals (*n* = 8) in both conditions are not able to (B). In the WT control group (*n* = 10), in the WT group that received IN OXT (*n* = 10), and in the APPswePS1dE9 group that received IN OXT (*n* = 8), object memory was found to be intact in a 1 h interval OLT (two-way ANOVA, Tukey *post-hoc* analysis) (C). In the Y maze there was no difference in arm entries between groups (D). In the Y-maze the WT control group (*n* = 10), the WT group that received IN OXT (*n* = 10), and the APPswePS1dE9 group that received IN OXT (*n* = 8) showed well-functioning working memory (one sample *t*-test against a baseline value of 50% alternations) (E). In the sociability assessment, AD OXT animals were less social than AD CTL animals and their respective WT counterparts (F). Bars represent mean±SEM. OLT, object location task; AD, Alzheimer’s disease; OXT, oxytocin; d2, discrimination index, ***p < 0.0005; **p < 0.005; ns, *p* > 0.05; ^#^*p* < 0.05 against 0.

To investigate if oxytocin treatment can reverse memory loss, we first established that AD animals were showing memory loss in an OLT setting. Before treatment, AD animals showed memory impairment, as they were unable to distinguish between the old and new position of an object in the OLT, while WT animals could distinguish (Fig. 1A). (One-sample *t*-test against 0 WT: *t* = 3.24, *p* = 0.0049, d2 = 0.27, SEM = 0.08; AD: *t* = 1.20, *p* = 0.25, d2 = 0.11, SEM = 0.092). At the half-way point of treatment performance between groups did not differ (multiple comparisons: WT CTL versus WT OXT: *p* = 0.99; WT CTL versus AD OXT: *p* = 0.69; WT OXT versus AD OXT: *p* = 0.70; WT CTL versus AD CTL: *p* = 0.20, AD OXT versus AD CTL: *p* = 0.99 WT OXT versus AD CTL: *p* = 0.53) (Fig. 1B). At the half-way point of treatment WT animals were able to distinguish between old and new position while AD animals could not (One-sample *t*-test against 0 WT CTL: *t* = 8.62, *p* = 0.0001, d2 = 0.34, SEM = 0.04; WT OXT: *t* = 2.97, *p* = 0.016, d2 = 0.26, SEM = 0.09; AD CTL: *t* = 0.90, *p* = 0.40, d2 = 0.10, SEM = 0.11; AD OXT: *t* = 0.98, *p* = 0.36, d2 = 10, SEM = 0.10).

After treatment, WT CTL, WT OXT, and AD OXT treated animals outperformed AD CTL animals significantly (Two-way ANOVA Interaction/Row/Column factor: *F* = 8.43/8.34/19.82; multiple comparisons: WT CTL versus AD CTL: *p* = <0.0001; WT OXT versus AD CTL: *p* = <0.0001; AD OXT versus AD CTL: *p* = 0.0026). There was no significant difference between WT CTL, WT OXT, and AD OXT treated animals (multiple comparisons: WT CTL versus WT OXT: *p* = >0.99; WT CTL versus AD OXT: *p* = 0.69; WT OXT versus AD OXT: *p* = 0.70) (Fig. 1C). After treatment, WT CTL, WT OXT, and AD OXT-treated mice were able to distinguish between the new and familiar position of two identical objects as indicated by the discrimination index (Fig. 1C) (one-sample *t*-test against 0 WT CTL: *t* = 8.08, *p* = 0.0001 d2 = 0.38 SEM = 0.03; WT OXT: *t* = 10.57, *p* = 0.0001, d2 = 0.38, SEM = 0.036; AD OXT: *t* = 2.58, *p* = 0.037, d2 = 0.29, SEM = 0.11). AD CTL mice did not remember the original position of the objects and explored both objects equally (one-sample *t*-test against 0 AD CTL: *t* = 1.67, *p* = 0.14, d2 = – 0.083, SEM = 0.05). Furthermore, there was a significant difference in exploration time between groups and multiple comparisons revealed a significant effect between WT OXT and AD CTL treated animals (Two-way ANOVA Interaction / Row / Column factor: *F* = 0.49 / 0.03 / 0.014; multiple comparisons: WT OXT versus AD CTL: *p* = 0.01; WT CTL versus WT OXT: *p* = 0.13; WT CTL versus AD CTL: *p* = 0.51; WT CTL versus AD OXT: *p* = 0.99) (Fig. 1G).

To strengthen the claim that intranasal oxytocin treatment restored memory we decided to investigate a different domain of memory, namely working memory by means of the Y-maze.

There was no difference in total arm entries across conditions (Two-way ANOVA Interaction/Row/Column factor: *F* = 0.49/0.03/0.01, *p* = 0.07/0.011/0.80) (Fig. 1D).

There was no significant difference between groups (Two-way ANOVA Interaction/Row/Column factor: *F* = 2.47/0.71/0.16; multiple comparisons: WT OXT versus AD OXT: *p* = 0.99; AD CTL versus AD OXT: *p* = 0.38; WT OXT versus AD CTL: *p* = 0.38; WT CTL versus WT OXT: *p* = 0.95; WT CTL versus AD CTL: *p* = 0.16; WT CTL versus AD OXT: *p* = 0.99) (Fig. 1E).

WT CTL, WT OXT as well as AD OXT mice showed intact working memory, as the percentage of alternations was significantly higher than 50% (one-sample *t*-test to 50% WT CTL: *t* = 5.58, *p* = 0.0003; WT OXT: *t* = 4.94, *p* = 0,0008; AD OXT: *t* = 47, *p* = 0.0023). AD CTL animals showed impaired working memory (one sample *t*-test to 50% AD CTL: *t* = 0.95467, *p* = 0.38) (Fig. 1E).

### Intranasal oxytocin administration decreases sociability in female APPswePS1dE9 mice

As oxytocin is associated with social memory and social behavior, we tested the effects of intranasal oxytocin administration on sociability by investigating how much time oxytocin-treated animals would spend with a new mouse over an empty compartment. AD OXT mice spent significantly less time exploring the new mouse, than WT CTL mice (Fig. 1C) (two-way ANOVA: Interaction *F* = 1.51, *p* = 0.23; row-factor *F* = 15.62, *p* = 0.0005; column-factor *F* = 11.47 *p* = 0.0021; WT CTL versus AD OXT: *p* = <0.0001) and AD CTL mice (AD CTL versus AD OXT: *p* = <0.01), as well as WT OXT mice (WT OXT versus AD OXT: *p* = <0.02). There was no difference in sociability between WT CTL, WT OXT, and AD CTL mice (WT CTL versus WT OXT: *p* = 0.24; WT CTL versus AD CTL: *p* = 0.37).

### Intranasal oxytocin administration does not affect hippocampal Aβ plaque load

**Fig. 2:**
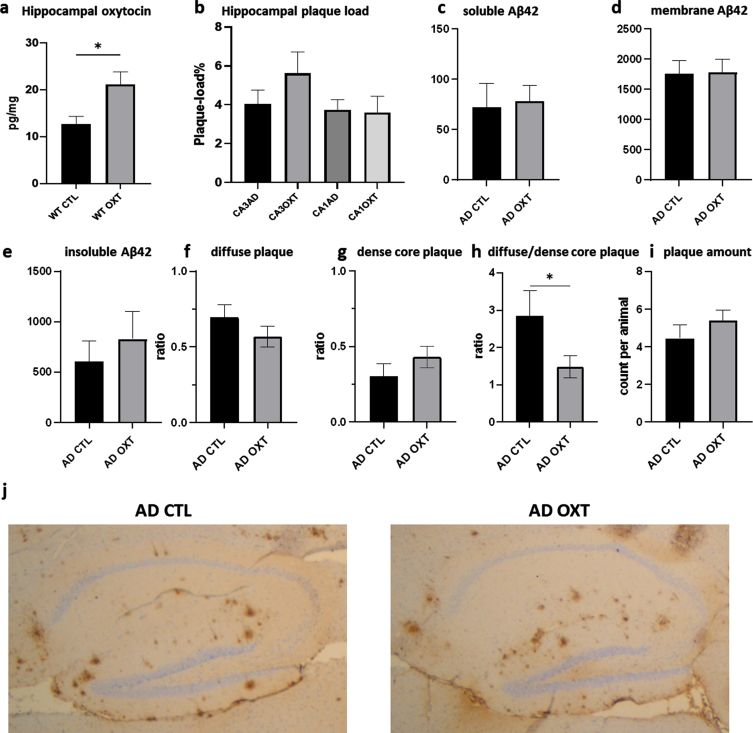
Intranasal administration of oxytocin increases hippocampal oxytocin levels in WT animals (a) does not alter the hippocampal plaque load in APPswePS1dE9 mice treated with oxytocin(b-i). Animals treated with oxytocin show a significant increase of hippocampal oxytocin levels over animals treated with saline (e) (Mann-Whitney test: WT CTL versus WT OXT *p* = 0.02 *n* = 8) (a). Aβ plaque load was quantified at 20x magnification in the hippocampus of APPswePS1dE9 mice by immunohistochemically staining Aβ (*n* = 8). Plaque load was calculated as the percentage of the total hippocampal surface area. Plaque load was not found to be altered within the hippocampus (*p* = 0.07) after oxytocin administration (Mann-Whitney test) (b). Hippocampal Aβ_42_ plaque-load by means of an ELISA showed no effect in soluble (c), membrane-associated (d) and insoluble fraction (e) of Aβ_42_ in AD animals. There was no observable difference in diffuse or dense core plaques or plaque amount between AD CTL and AD OXT (f-g). There are more diffuse plaques compared to dense plaques in AD CTL compared to AD OXT animals (*p* = 0.04) (h). Representative images of the Aβ-stained hippocampus and cortex for the APPswePS1dE9 control group, the APPswePS1dE9 oxytocin-treated group are shown at 2.5x magnification (j). Bars represent mean±SEM. Aβ, amyloid-β; AD, Alzheimer’s disease; WT, wild type. *p < 0.05.

To investigate if intranasal oxytocin administration affected plaque load, we stained for Aβ_42_, revealing no differences between oxytocin- and vehicle-treated groups (Fig. 2b) (Mann-Whitney test: *p* = 0.0754 *n* = 8). Analysis of hippocampal Aβ_42_ plaque-load by means of an ELISA also revealed no effect of oxytocin treatment for soluble Aβ_42_ (Fig. 2c) (Mann-Whitney test: *p* = 0.62, *n* = 8), membrane-associated Aβ_42_ (Fig. 2d) (Mann-Whitney test: *p* = 0.96, *n* = 8) or the insoluble Aβ_42_ fraction (Fig. 2e) (Mann-Whitney test: *p* = 0.44, *n* = 8). There was no difference in diffuse (Fig. 2f) (Mann-Whitney test: *p* = 0.24, *n* = 8) and dense core plaques (Fig. 2) (Mann-Whitney test: *p* = 0.25, *n* = 8). AD CTL animals showed significantly more diffuse plaques than dense core plaques than AD OXT animals. There was no difference in amount of plaques (Mann-Whitney test: *p* = 0.33, *n* = 8) (Fig. 2i) between oxytocin and vehicle-treated groups.

#### Intranasal oxytocin administration increases hippocampal oxytocin

To assess whether intranasal oxytocin treatment actually ended up in the hippocampus, we investigated the amount of hippocampal oxytocin would increase in WT animals after receiving treatment. Intranasal oxytocin administration increased hippocampal oxytocin as indicated by ELISA (Fig. 2e).

### mTOR is upregulated in female APPswePS1dE9 mice treated with oxytocin

**Fig. 3:**
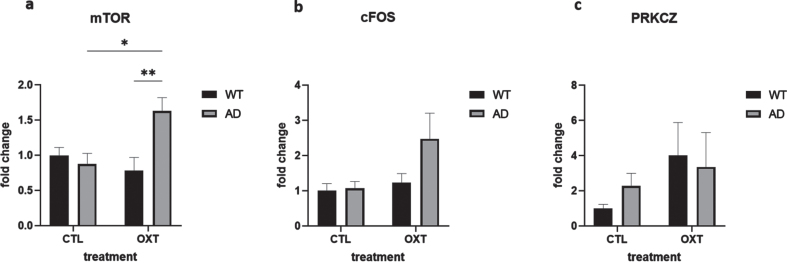
Intranasal administration of oxytocin in an AD model increases expression of *mTOR*. The expression of several mediators in the oxytocin-OXTR pathway was measured in hippocampal tissue isolated from oxytocin- or vehicle-treated AD and wild type mice after 42 days of treatment. Expression was normalized using *GAPDH* and *HPRT1* and converted to fold change values (as compared to WT control animals). Expression of *mTOR* was upregulated in the AD group that received oxytocin (*n* = 8) compared to the AD control group (a) (*n* = 8) (two-way ANOVA with Tukey *post-hoc* analysis). Expression of cFOS and PRKCZ was not significantly different in any condition (b, c). Bars represent mean±SEM. mTOR, mammalian target of rapamycin; PKM*ζ*, protein kinase M *ζ*; AD, Alzheimer’s disease; OXTR, oxytocin receptor; GAPDH, glyceraldehyde-3-phosphate dehydrogenase. **p < 0.005, *p < 0.05.

To investigate potential mechanisms of actions of intranasal oxytocin treatment we analyzed the downstream targets of the oxytocin pathway via qPCR analysis. Analysis revealed that mammalian target of rapamycin (mTOR) was upregulated in oxytocin-treated APPswePS1dE9 mice when compared to vehicle-treated APPswePS1dE9 mice and wild-type oxytocin-treated animals (Fig. 3a) (Two-way ANOVA Interaction/Row/Column factor: *F* = 8.93/ 2.73/4.96; multiple comparisons: WT OXT versus AD OXT: *p* = 0.0061; AD CTL versus AD OXT: *p* = 0.0162; WT OXT versus AD CTL: *p* = 0.98; WT CTL versus WT OXT: *p* = 0.77; WT CTL versus AD CTL: *p* = 0.95; WT CTL versus AD OXT: *p* = 0.05).

cFOS expression was not significantly changed (Fig. 3b) (Two-way ANOVA Interaction/Row/Column factor: *F* = 2.36/4.67/2.94; multiple comparisons: WT OXT versus AD OXT: *p* = 0.13; AD CTL versus AD OXT: *p* = 0.07; WT OXT versus AD CTL: *p* = 0.98; WT CTL versus WT OXT: *p* = 0.97; WT CTL versus AD CTL: *p* = 0.99; WT CTL versus AD OXT: *p* = 0.054).

PRKCZ expression was not affected either (Fig. 3c) (Two-way ANOVA Interaction/Row/ Column factor: *F* = 0.47/2.1/0.05; multiple comparisons: WT OXT versus AD OXT: *p* = 0.99; AD CTL versus AD OXT: *p* = 0.96; WT OXT versus AD CTL: *p* = 0.82; WT CTL versus WT OXT: *p* = 0.38; WT CTL versus AD CTL: *p* = 0.92; WT CTL versus AD OXT: *p* = 0.65).

### Oxytocin increases neurite growth in vitro

**Fig. 4:**
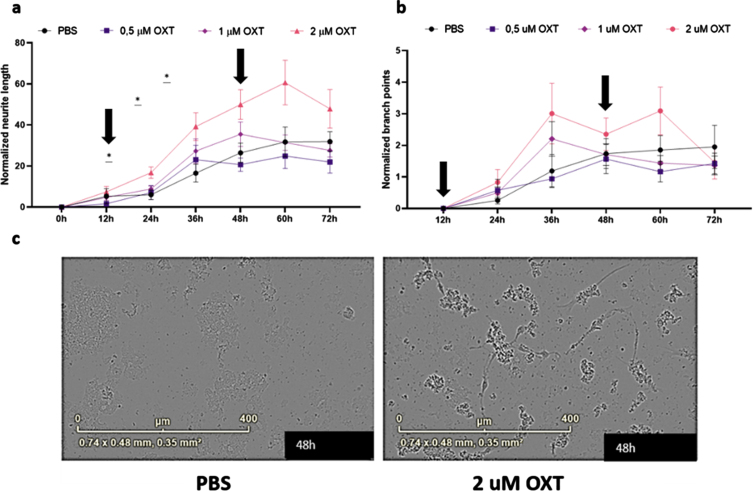
Oxytocin promotes neurite outgrowth, but not branch point formation, in maturing primary rat neurons *in vitro.* Neurite length per area (a) and the number of branch points per area (b) were evaluated using live-cell imaging. Every 3 h, an analysis was performed. Subsequently, averages were taken per 12 h, which were normalized against the 0 h time point values of the PBS control group (*n* = 9). In the presence of 2*μ*M oxytocin (*n* = 9), neurite length was increased from 24 h (*p* = 0.025) up to 48 h (*p* = 0.041), after which the beneficial effect gradually diminished. Both incubation time and oxytocin concentration had a significant effect on neurite length. Oxytocin concentrations of 0.5*μ*M (*n* = 9) and 1*μ*M (*n* = 9) did not significantly affect neurite length, and neither of the concentrations did increase branch point formation in comparison to the PBS control group. Differentiated HT22 cells do not show increased neurite length when treated with oxytocin regardless of presence or absence of Aβ_42_. Black arrows indicate initial addition of oxytocin to medium as well as boost after 48 h (a, b). Representative images for primary neurons after 48 h of treatment with 2*μ*M of OXT (left) and PBS (right) (c). One-way ANOVA and Tukey *post-hoc* analysis. Data points represent mean±SEM. *p < 0.05.

To assess the effects of oxytocin treatment *in vitro*, we treated maturing primary rat cortical neurons with 0.5– 2*μ*M oxytocin. Analysis of maturing primary rat cortical neurons revealed a significant treatment effect of oxytocin on normalized neurite length, but not on normalized branch points, indicating an increase of neurite growth of oxytocin treatment *in vitro* (Fig. 4a) (Two-way ANOVA Interaction/Row/Column: *F* = 2.16/56.98/3.78; *p* = 0.006/ < 0.0001/0.02).

## DISCUSSION

In this study, we aimed to investigate the effects of low dose (0.5*μ*g/kg) intranasal oxytocin treatment on memory and AD-relevant pathology in 9-month-old female APPswePS1dE9 mice. Our results show that treatment with oxytocin had beneficial effects on memory, reversing cognitive decline, as indicated in both the OLT and the Y-maze test. Prior to treatment, at 8 months of age, female APPswePS1dE9 mice were unable to remember the location of an object in the OLT. After 2 weeks of treatment, we could not see any effect of oxytocin on memory as indicated by the OLT. Yet, after 4 weeks of oxytocin treatment, female APPswePS1dE9 mice were able to remember the location of an object (OLT), and showed intact working memory (Y-maze), comparable to WT animals. Vehicle-treated female APPswePS1dE9 mice were unable to do either. Interestingly, the results on the sociability assessment showed the opposite pattern, with vehicle-treated female APPswePS1dE9 mice showing more social behavior comparable to WT animals, whereas oxytocin-treated female APPswePS1dE9 mice showed decreased social behavior. This is in line with research showing chronic administration of oxytocin to decrease sociability in voles, where chronic infusion of oxytocin was shown to decrease preference of interacting with a novel social stimulus over a novel object [36].

As our results showed, analyzing the effects of oxytocin treatment on the neurobiology of AD revealed that oxytocin did not significantly influence Aβ_42_ plaque load. However, AD CTL animals showed a higher ratio of diffuse plaques compared to dense core plaques than AD animals treated with oxytocin. This is consistent with research from Lemke et al. indicating dense core plaques to show neuroprotective properties mediated by microglia [37].

Importantly, long-term intranasal oxytocin treatment increased oxytocin levels in the hippocampus. We suggest a direct correlation between increased oxytocin in the hippocampus and improved memory of APPswePS1dE9 mice, as oxytocin signaling is involved in memory, which should be investigated in the future. The exact mechanism as to how intranasal treatment using oxytocin reverses cognitive decline in APPswePS1dE9 mice is still to be elucidated. Given how the effect of oxytocin treatment on memory was observable after 4 weeks of treatment, it is unlikely that the effect of treatment was due to an acute effect.

To investigate potential mechanisms by which oxytocin restores cognitive function in APPswePS1dE9 mice, we examined hippocampal PRKCZ signaling, as PRKCZ is involved in establishing long-term memories. PRKCZ expression was not significantly increased in oxytocin-treated APPswePS1dE9 mice though. Notably, qPCR analysis revealed an increase of hippocampal mTOR mRNA levels in oxytocin-treated APPswePS1dE9 mice. mTOR upregulation has been associated with long-term memory retrieval, as the mTORC1 inhibitor rapamycin leads to long-term, but not short-term, memory impairment [38]. Interestingly, mTOR upregulation has been implicated in contributing to AD pathology, often by inhibiting autophagy, blocking a cell’s ability to remove protein aggregates [39]. While c-Fos and PRKCZ mRNA levels were not significantly different across conditions, we do see a clear trend of c-Fos mRNA expression in oxytocin treated AD animals. C-Fos is involved in proliferation and differentiation during development [40]. C-Fos mRNA expression has been shown before to be upregulated in AD, considered as a compensatory mechanism against neuronal loss. C-Fos is seen as a marker of neuronal activity and oxytocin can upregulate c-Fos via ERK5 binding [41]. Another mechanism mediating the cognitive effects of oxytocin could involve an inhibition of the ERK1/2 pathway, which has also been implicated in AD [42]. Interestingly, microinjections of oxytocin into the male rat PVN have been shown to upregulate phosphorylation of ERK in the hypothalamus in a dose-dependent manner, with an increase leading to more phosphorylation of ERK1/2 [43]. However, low doses of intranasal administration of oxytocin (1.25 IU) were shown to lead to decreased hippocampal ERK1/2 activation in a female rat model of AD [29]. Whether or not these seemingly contradicting findings are to be explained in view of the method of administration, the dose, the brain regions(s) assessed, sex or genotype of the animals remains to be elucidated in order to reach a better understanding of the effect of oxytocin in the brain in general [29]. Men and women suffering from AD exhibit different cognitive as well as psychiatric symptoms. Women show faster decline after diagnosis of MCI and more rapid brain atrophy in comparison to men [44]. The fact that AD manifests itself differently in humans, but also rodents based on sex, as insoluble and soluble Aβ levels are increased in female over male mice is important to consider [45]. It is possible that the effects of oxytocin on memory are sex-specific. As such, more research is necessary to replicate these findings in a male APP/PS1 cohort.

Our results suggest oxytocin increases neurite growth *in vitro.* Increased neurite growth is associated with improved CNS regeneration [46]. As oxytocin treatment did not modulate Aβ_42_ plaque load, it is likely that the effects of oxytocin treatment *in vitro* and *in vivo* are not disease– modifying, but beneficial to memory more generally, acting as a cognition enhancer where cognitive deficit is present, independent of Aβ pathology. It is interesting to note that memory of WT treated animals with oxytocin did not improve. This indicates that there needs to be a memory deficit present that oxytocin signaling in turn can improve. This is also evident by the fact that mTOR as well as c-Fos mRNA expression are only increased in oxytocin treated AD animals, but not oxytocin treated WT animals.

While the role for oxytocin in memory has been investigated and evidence suggests oxytocin to upregulate LTP, surprisingly, it has not been considered as a treatment against AD until recently. As stated earlier, El-Ganainy et al. showed that long-term intranasal oxytocin treatment is able to ameliorate symptoms of AD in an aluminum-chloride induced model in female rats [29]. While this pharmacological model differs from the APPswePS1dE9 transgenic mouse model used here, it is relevant as translational results are highly desirable. It is interesting to point out that while El-Ganainy and colleagues have shown Aβ_42_ levels to be downregulated upon intranasal treatment with oxytocin, we could not observe such a change in Aβ_42_ levels in our study. Next to the different nature of the model used, the difference in observations can be potentially explained by the method of measuring Aβ_42_, as we separated Aβ_42_ phases to obtain the insoluble fraction of Aβ_42_, whereas El-Ganainy et al. investigated total fibrillary Aβ_42_ levels. Currently, it is unclear as to the mechanism by which oxytocin decreases Aβ_42_ levels. It is important to note that modulating plaque load is not essential to have an impact on cognition [31, 34].

In a recent interesting study by Selles et al., it was shown that oxytocin was able to attenuate microglial activation and restore social and non-social memory in APPswePS1dE9 mice. Interestingly, while oxytocin seems to restore memory in APPswePS1dE9 mice in the present study, our results showed APPswePS1dE9 mice treated with oxytocin to be less social to an unfamiliar conspecific than either WT or APPswePS1dE9 mice treated with saline [30].

Oxytocin is an FDA-approved drug and currently used in human trials in different contexts, such as autism or schizophrenia [47– 50]. The current research shows the potential of low-dose oxytocin treatment against AD. Future experiments should aim to elucidate the working mechanisms of low-dose intranasal oxytocin treatment on AD to optimize treatment options better and to investigate potential peripheral effects.

## Supplementary Material

Supplementary Material

## Data Availability

The data supporting the findings of this study are available on request from the corresponding author. The data are not publicly available due to privacy or ethical restrictions.
