# Supplementary material for: Exogenous Oxytocin Administration Restores Memory in Female APP/PS1 Mice
Source: J Alzheimers Dis. 2023 Nov 21;96(3):1207–19. doi: 10.3233/JAD-230657 (PMC10741313; doi:10.3233/JAD-230657)
Supplement: Supplementary Material [file jad-96-jad230657-s001.pdf]

Supplementary Material

Exogenous Oxytocin Administration Restores Memory in Female APP/PS1 Mice

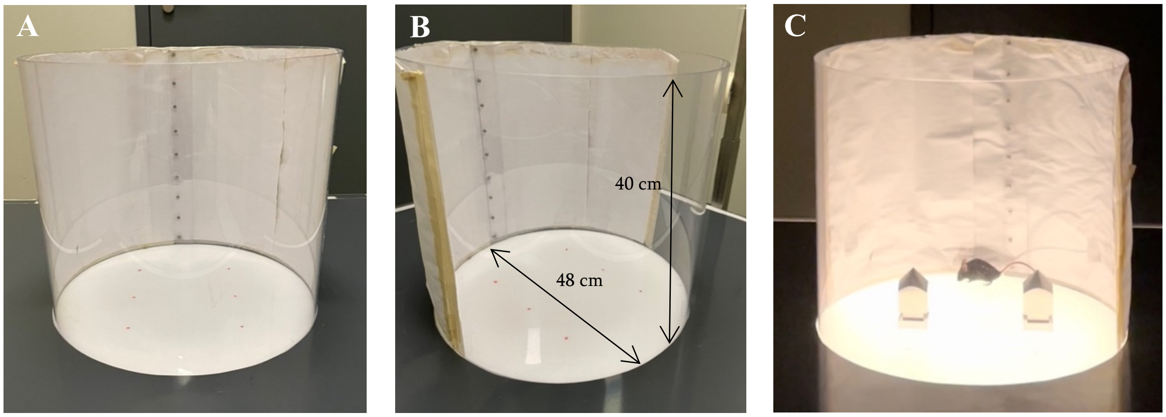

Supplementary Figure 1. Arena used in the object location task. This cylinder-shaped plastic arena has a diameter of 48 cm and a height of 40 cm (A, B). The back half of the plastic is covered in white paper, while the front half is transparent. The base surface of the arena is a light source (C).

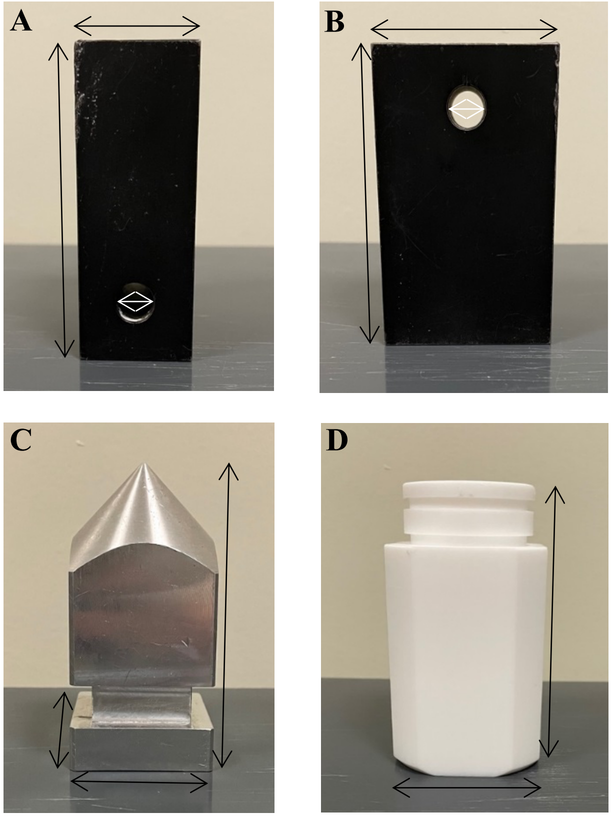

Supplementary Figure 2. Objects used in the object location task. Three different shapes of objects were used and randomized during testing.

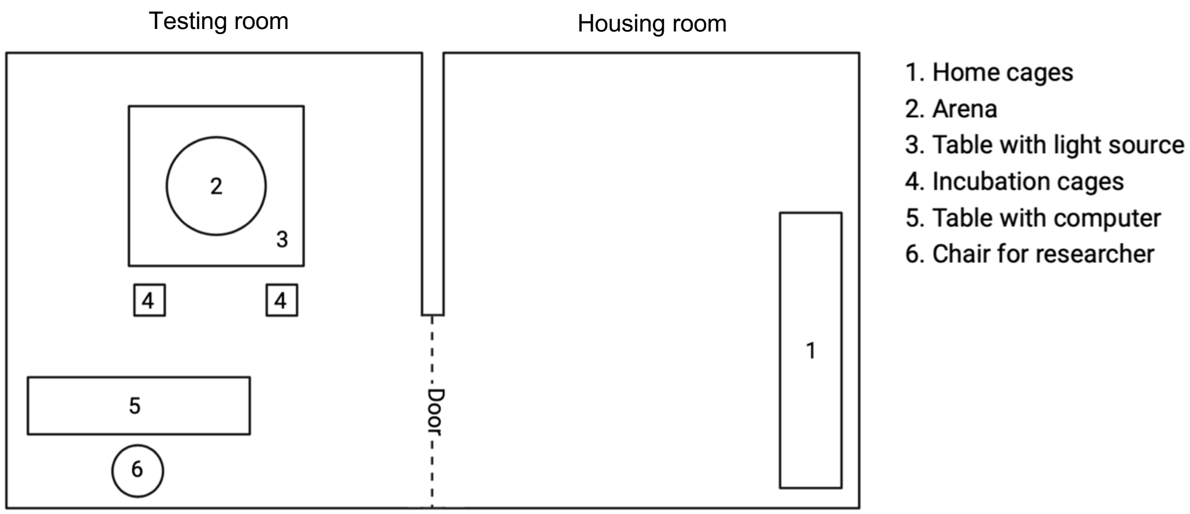

Supplementary Figure 3. Set up of the rooms where the object location task was performed. The animals were housed in a room separate from the testing room. The testing room included two incubation cages.

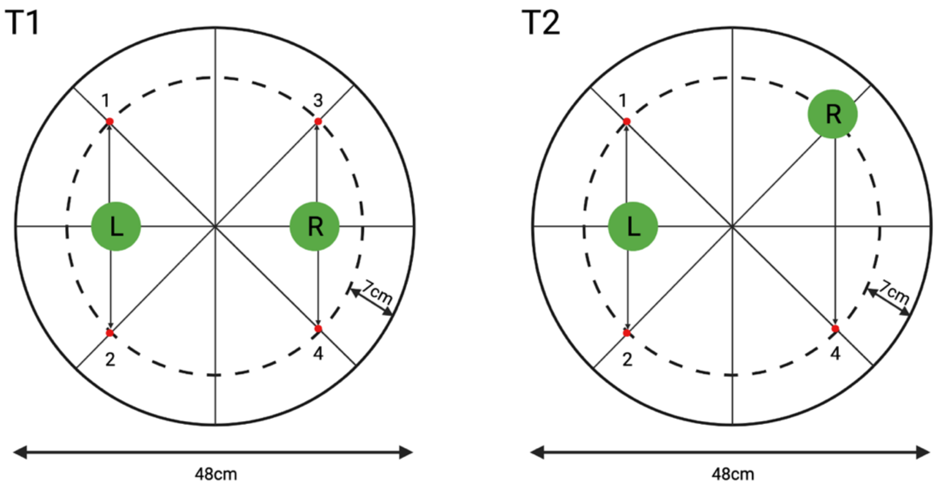

Supplementary Figure 4. Configuration of the arena in trial 1 (T1) and trial 2 (T2). During T1, two identical objects were placed at the midline of the arena. After 3 min of exploration, the animal was returned to its home cage. After a 1 h delay, the animal was placed back into the arena for T2, where either the left (L) or right (R) was moved to a different position within the arena.

Y maze spontaneous alternations task

A Y maze spontaneous alternations task was performed to evaluate spatial working memory after 35 days of treatment. The Y maze has three similar arms (A, B, and C) that are all placed 120° from each other (Supplementary Figure 5). The maze is made from grey, non-reflective material. Each arm is 40 cm long and contains a visual cue, which in our case, were pieces of colored tape. Underneath the arena, an indirect source of light was placed to enable the researcher to observe the animal. The setup of the testing room and housing room are similar to the one displayed in Supplementary Figure 3, with the OLT arena being replaced by the Y maze. During this one-trial test, the mice were able to freely explore the maze for 6min. About 5min before their trial, mice were taken from their home cages and kept in an incubation cage inside the testing room, but never facing the maze. Mice were always placed into the arena facing the center of the maze and in a specific order (i.e., first mouse in arm A, second mouse in arm B, third mouse in arm C, etc.). Arm entries were manually registered and considered valid when both hind paws were entirely inside the arm. If the mouse entered three different arms consecutively, this was counted as a triad. The percentage of alternations was calculated (alt% = number of triads / (number of entries - 2) * 100) as a measure for working memory. If the calculated % alternations is significantly over 50%, the mouse shows well-functioning working memory. In between different mice, the maze was cleaned with 70% ethanol in order to avoid the presence of olfactory cues.

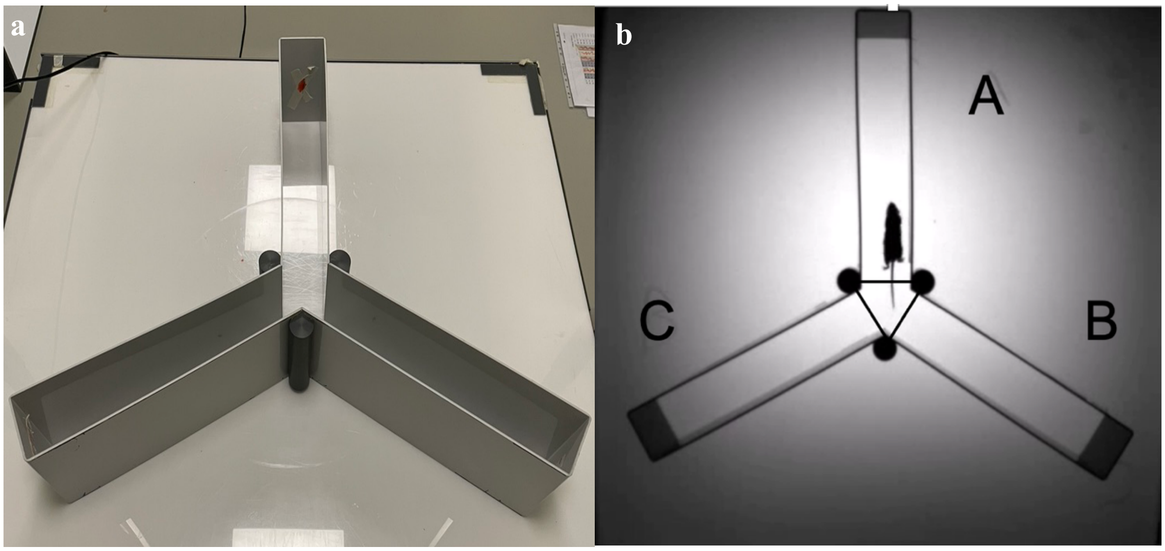

Supplementary Figure 5. Y maze spontaneous alternations task. The Y maze has three similar arms (A, B, and C) that are all placed 120° from each other. The maze is made from 40cm long plastic grey arms, with each arm containing a visual cue (a). The base surface of the maze is a light source (b).

Sociability assessment

A three-chamber sociability assessment was performed to evaluate cognition in the form of general sociability. The arena consists of 3 chambers, each having an area of 20 x 16 x 25 cm. The two outer chambers contain a metal cage. The arena's walls are made from Plexiglas and the bottom is white (Supplementary Figure 6). Underneath the arena, an indirect source of light was placed to enable the researcher to observe the animal. The setup of the testing room and housing room are similar to the one displayed in Supplementary Figure 3, with the OLT arena being replaced by the sociability arena. One of those cages contained a social target being an unknown mouse, while the other cage was empty. About 5min before their trial, mice were taken from their home cages and kept in an incubation cage inside the testing room, but never facing the arena. Mice were introduced in the middle chamber of the arena facing the wall and were able to explore for 10 min. Sociability was scored as the relative exploration time spent in the chamber with the social target, expressed as the discrimination index (d2): (d2 = [(exploration time social target) − (exploration time empty chamber)] / (total exploration time)). In between different mice, the maze was cleaned with 70% ethanol in order to avoid the presence of olfactory cues.

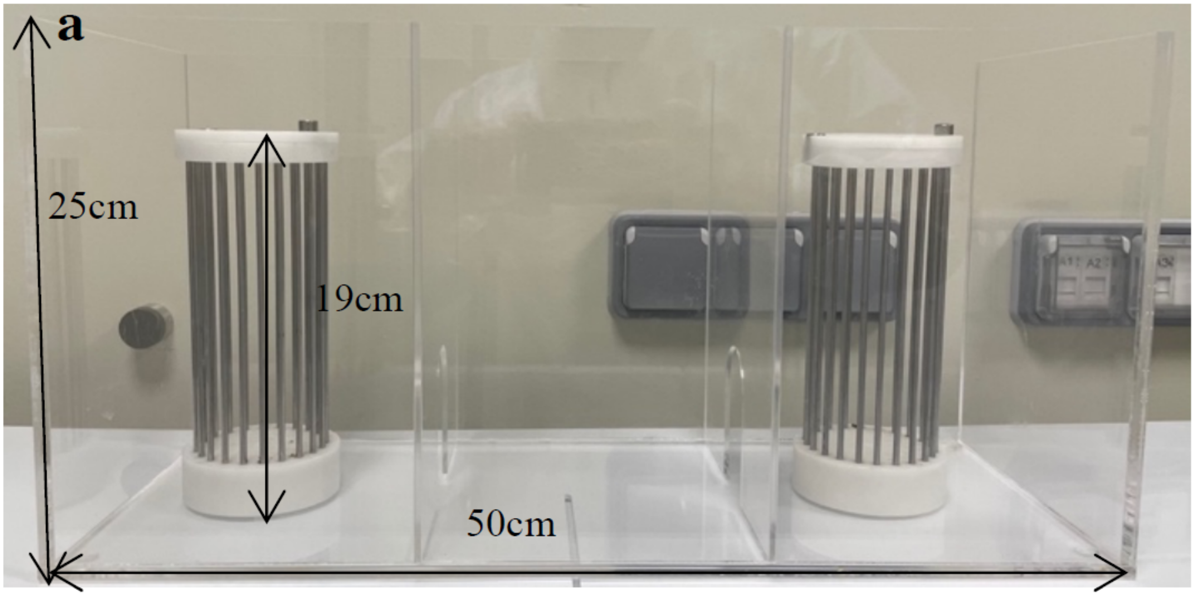

Supplementary Figure 6. Sociability arena. The arena consists of 3 chambers, each having an area of 20 x 16 x 25 cm. The arena's walls are made from Plexiglas and the bottom is white.

Real-time qPCR

Supplementary Table 1. qPCR primer pair sequences.

Gene	Forward primer sequence	Reverse primer sequence
PKMζ<br>*Protein kinase M zeta*	5' - CCTTCTATTAGATGCCTGCTCTCC - 3'	5' - TGAAGGAAGGTCTACACCATCGTTC - 3'
mTOR<br>*Mechanistic target of rapamycin*	5' - TGTCTGATTCTCACCACGCA - 3'	5' - CTCTTTGGCCAGGGTCTCAT - 3'
cFOS	5' - ACCATGATGTTCTCGGGTTTCAA - 3'	5' - GCTGGTGGAGATGGCTGTCAC - 3'

Supplementary Table 2. Housekeeping genes used for qPCR analysis against whose levels all experimental genes were normalized.

Tissue type/cell type	First housekeeping gene	Second housekeeping gene
Hippocampus	*glyceraldehyde 3-phosphate dehydrogenase (GAPDH)*	*hypoxanthine-guanine phosphoribosyltransferase (HPRT1)*
